# Corneal Lymphangiogenesis: Current Pathophysiological Understandings and Its Functional Role in Ocular Surface Disease

**DOI:** 10.3390/ijms222111628

**Published:** 2021-10-27

**Authors:** Hyung-Keun Lee, Sang-Mok Lee, Dong-Ihll Lee

**Affiliations:** 1 Department of Ophthalmology, Institute of Vision Research, Yonsei University College of Medicine, Seoul 06273, Korea; 2 Department of Ophthalmology, HanGil Eye Hospital, Catholic Kwandong University College of Medicine, Incheon 21388, Korea; lsm10003@gmail.com; 3 Medical School, Capital Medical University, Beijing 100069, China; seananners73@gmail.com

**Keywords:** lymphangiogenesis, allograft rejection, dry eye disease, lymphatic endothelium, vascular endothelial growth factor (VEGF)

## Abstract

The cornea is a transparent and avascular tissue that plays a central role in light refraction and provides a physical barrier to the external environment. Corneal avascularity is a unique histological feature that distinguishes it from the other parts of the body. Functionally, corneal immune privilege critically relies on corneal avascularity. Corneal lymphangiogenesis is now recognized as a general pathological feature in many pathologies, including dry eye disease (DED), corneal allograft rejection, ocular allergy, bacterial and viral keratitis, and transient corneal edema. Currently, sizable data from clinical and basic research have accumulated on the pathogenesis and functional role of ocular lymphangiogenesis. However, because of the invisibility of lymphatic vessels, ocular lymphangiogenesis has not been studied as much as hemangiogenesis. We reviewed the basic mechanisms of lymphangiogenesis and summarized recent advances in the pathogenesis of ocular lymphangiogenesis, focusing on corneal allograft rejection and DED. In addition, we discuss future directions for lymphangiogenesis research.

Lymphatic vessels (LVs) form capillary-like networks and play an essential role in maintaining fluid balance in the body; they are involved in the pathogenesis of many diseases including ocular diseases. Lymphangiogenesis, the formation of new lymphatic vessels from pre-existing vessels, is rare under normal conditions but frequently occurs under pathological conditions, including inflammation, tissue repair, and tumor growth [1]. In this review, we summarize recent progress in the field of corneal lymphangiogenesis, focusing on corneal allograft rejection, dry eye disease (DED), and other diseases, including herpes simplex virus (HSV) keratitis and allergies.

## 1. Cytokines and Molecular Markers for Lymphangiogenesis

Under normal conditions, both the peripheral and central cornea have a specific structure for maintaining transparency with limited blood and lymphatic vessels. Inflammatory damage can lead to the invasion of blood and lymphatic vessels into the cornea. Normal blood vessels (BVs) may contain smooth muscle cells or pericytes that cover the vascular endothelium, while lymphatic capillaries are lined with a continuous single cell layer of endothelial cells and have discontinuous basement membranes, and are not encircled by pericytes or smooth muscle cells [1,2].

During embryogenesis, the LV first appears at embryonic day 9.5 (E9.5) when blood circulation is established [3]. The progenitors of lymphatic endothelial cells (LECs) express the transcription factor prospero homeobox protein 1 (PROX1) and then begins to migrate away from the vascular system, forming the first primitive lymphatic vessel, the dorsal peripheral longitudinal vessel in the jugular region of the embryo. The second population of cells form lymph sacs (also called the ventral primordial thoracic duct) that expand further by lymphangiogenic sprouting [4,5]. In addition to the venous source, alternative cellular origins for LECs have been described in the skin and heart [5]. In the skin, LEC progenitors are derived from the vascular endothelium, which forms primitive lymphatic clusters that eventually fuse with LVs of venous origin [6].

Despite the development and progress in lymphangiogenesis research, it is still not easy to identify LECs. Molecular markers for LECs widely used for LEC and lymphangiogenesis research include PROX1, lymphatic vessel hyaluronan receptor (LYVE)-1, EphrinB2, and Podoplanin [2,7,8]. However, to the best of our knowledge, there are no known specific markers for LECs. Therefore, to specifically identify LECs, a combination of the aforementioned markers is generally used.

Several cytokine families have been implicated in lymphangiogenesis and can be classified into two groups. The first group includes the vascular endothelial growth factors C and D (VEGF-C and -D) and angiopoietins (ANGs) that act directly on LECs. The others, which are mainly inflammatory cytokines, including interleukin (IL)-1, IL-17, and tumor necrosis factor (TNF)-α, may act indirectly through the regulation of VEGF-C expression. Therefore, as in hemangiogenesis, the VEGF family is the key regulator of LV development in embryogenesis and lymphangiogenesis in adults, through activation of VEGF receptor-3 (VEGFR-3). Before reviewing ocular lymphangiogenesis and its pathological role, some important cytokines and their activation systems for lymphangiogenesis are briefly reviewed.

### 1.1. Vascular Endothelial Growth Factor (VEGF)s; Focused on VEGF-C and -D

**Figure 1:**
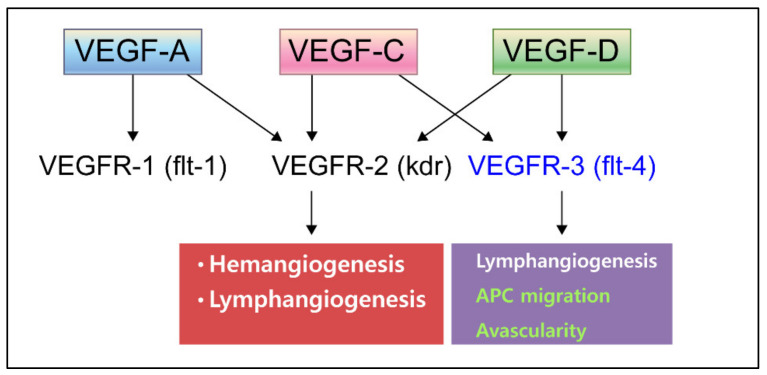
Schematic illustration of VEGFs and their receptor system.

VEGFs are essential cytokines for the development and maintenance, of both vascular and lymphatic endothelial cells (VECs and LECs). It is well known that the gradient of VEGFs that form by their interaction with proteoglycan [7] and co-receptors such as neuropilin 1 and 2 (NRP1–2) [8,9] induce endothelial cell (EC) activation, determine the polarity of the vascular sprout, and establishment of the mature vessel [7]. VEGFs and their receptors are complex and promiscuous ligand-receptor systems. VEGFs include five dimeric polypeptides—VEGF-A, VEGF-B, VEGF-C, VEGF-D, and placental growth factor (PGF). In vivo, in its active form, VEGFs are structurally homodimeric polypeptides of 45 kDa that undergo alternative splicing or proteolytic processing, regulating their binding to the three structurally related VEGF receptor tyrosine kinases (VEGFR1–3) [1,10]. Of particular importance for the regulation of LECs are VEGF-C and VEGF-D, which mainly bind to VEGFR3 and the co-receptor NRP2 [6]. However, VEGF-C and VEGF-D can also bind and activate VEGFR2 (Figure 1) [11,12].

**Figure 2:**
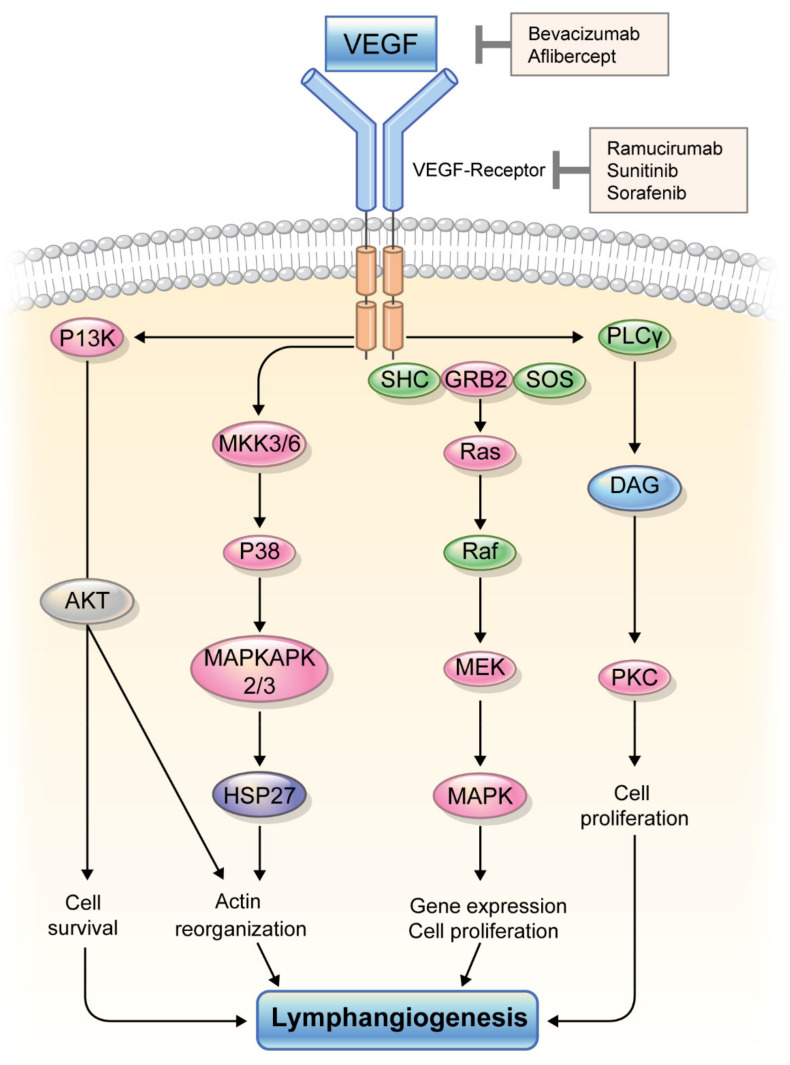
Intracellular signaling pathway of VEGF and VEGF receptor for lymphangiogenesis. VEGF, vascular endothelial growth factor.

The intracellular signaling pathway for VEGF-induced lymphangiogenesis involves VEGFR3 activation by binding VEGF-C resulting in the phosphorylation of protein kinase B (PKB)/AKT and extracellular regulated kinases (ERK)1/2 kinases, promoting LEC migration, proliferation, and survival [13]. In addition, phosphoinositide 3 kinase (PI3K), an upstream activator of AKT, is required for LEC tube formation and migration in vitro [14] (Figure 2). There is substantial evidence of defective lymphangiogenesis and vascular failure from gene inactivation of the above markers (e.g., VEGFR3) in genetically modified mouse model studies [1,15,16].

Similar to VEGF-C, VEGF-A activation of VEGFR2 results in lymphatic hyperplasia in adults [2,17]. In addition, specific deletion of *Vegfr2* in LECs leads to the formation of a hypoplastic but functional lymphatic vasculature [11]. Therefore, VEGF-A/VEGFR2 appears to partially influence lymphatic vessel growth and functional maintenance in healthy mice.

### 1.2. Angiopoietins (ANG)

**Figure 3:**
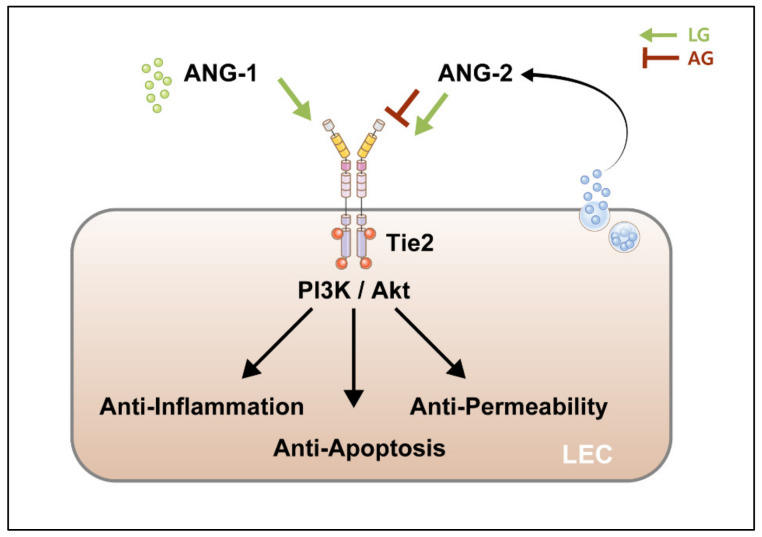
Schematic illustration of current understanding of angiopoietins and Tie receptor activation. ANG, angiopoietin; LEC, lymphatic endothelial cell; LG, lymphangiogenesis; AG, angiogenesis.

ANG, a ligand for the Tie2 and Tie2 receptors, plays an important role in both angiogenesis and lymphangiogenesis. ANG1 and ANG2 have an unusual structure with approximately 500 amino acid residues with predicted coiled-coil and fibrinogen-like domains [18]. The ligand-receptor interaction in the Ang/Tie is complicated, and the biological effect is different whether Tie1 exists in target cell (Figure 3). Generally, ANG1 and ANG2 exert their effects through a receptor tyrosine kinase, Tie2 (also known as Tek), expressed on ECs and perivascular cells [15]. Tie1, a close homolog of Tie2, is expressed on the vascular and lymphatic endothelium [16] that regulates the ability of ANG2 to activate Tie2. In the absence of Tie1, ANG2 acts as a Tie2 agonist, whereas in the presence of Tie1, it acts as a Tie2 antagonist.

Tie1 expression is relatively low in LECs [19] and thus ANG2 may work as a Tie2 agonist in the lymphatic system, similar to ANG1. ANG2 can bind Tie2 on adjacent cells and this complex is important for lymphatic junctional stability [20]. By blocking ANG2, intercellular integrity of LEC is reduced, and become leaky. However, ANG1/2-induced signal transduction and their biological effects on LECs remains to be elucidated.

It is well known that ANG1/2 are potent lymphangiogenic factors in the ocular surface. Using a corneal pocket assay, Morisada et al. found that ANG1 promotes LYVE-1 in LECs [21]. ANG1 and ANG2 were also found to be potent lymphangiogenic factors in an inflammatory angiogenesis model [22]. In addition to lymphangiogenesis, ANG1 also exerts vascular stability by recruiting tyrosine protein kinase-like 7 (PTK7)+ mononuclear cells, which may have a pericyte-like function [23]. Recently, Zhang and Chen [24] showed improved corneal allograft survival through ANG2 blockade, which reduced donor-derived cell trafficking to draining lymph node (LN).

The Ang/Tie system also has critical roles in Schlemm’s canal integrity and glacomatogenesis. Mice with deletion in either *Ang1* or *Ang2* lack aqueous drainage pathways and develop glaucoma [25,26].

### 1.3. Fibroblast Growth Factor2 (FGF2)

FGF2, known as basic FGF (bFGF), induces lymphangiogenesis in vivo, including in the cornea. Using the corneal micropocket assay, several studies have demonstrated that the effect of FGF2 is entirely dependent on VEGFC/VEGFR3 [27,28,29]. In addition to the FGF2 and VEGF system, the Notch/Dll4 pathway was shown to enhance lymphangiogenesis through activation of the bFGF system [30,31] or HIF-1α activation [32].

FGF2 can also be biologically neutralized by soluble LYVE-1, which forms a specific complex with FGF2 [33,34].

### 1.4. Hepatocyte Growth Factor (HGF), Platelet-Derived Growth Factors (PDGF), Insulin-Like Growth Factors (IGF), Endothelin, and Transforming Growth Factor (TGF)-β

Other growth factors with pro-lymphangiogenic activity have been identified through cancer research. These include HGF [35,36,37], PDGF [37,38,39], IGF [40,41], and endothelin-1 [42,43]. TGF-β has also reportedly regulated lymphatic vessel sprouting during development [44,45]. In the cornea, TGF-β-induced protein (TGFBIp), which is abundant in the cornea, enhances lymphangiogenesis; however, the underlying mechanisms remain unknown [46].

### 1.5. Other Factors That Induce Lymphangiogenesis

Several other factors have been reported to be involved in corneal lymphangiogenesis. However, most of them affect lymphangiogenesis by recruiting or activating immune cells that secrete VEGFs [47,48,49].

Substance P (SP) is a neuropeptide composed of 11 amino acids and is known to act as a key pro-inflammatory molecule in neurogenic inflammation; its release is induced by substances derived from afferent nerve endings upon nerve damage or stimulation [50]. SP exerts effects through high-affinity binding to neurokinin receptor-1 (NK1R), facilitating numerous physiological and pathological effector mechanisms, such as chemotaxis, vasodilation, cell migration/proliferation, differentiation into effector T cells, stimulation of mast cell degranulation, and angiogenesis/lymphangiogenesis [51,52]. SP-induced (lymph)angiogenesis has been reported for various corneal inflammatory conditions, but the mechanism by which SP induces (lymph)angiogenesis other than immune cell infiltration has not been well studied [53,54]. A recent study reported that, in in vitro and in vivo models, corneal lymphangiogenesis in DED is regulated by SP through the regulation of expression of VEGFR3 [50].

IL-17A (also known as IL-17), secreted by T helper cell type 17 (Th17), is a potent proinflammatory cytokine that plays an important role in the pathogenesis of various autoimmune diseases, including DED [55,56]. It is also known as an angiogenic factor that induces pathological angiogenesis and lymphangiogenesis. IL-17 is known to induce angiogenesis in malignancies [57], rheumatoid arthritis [58], and HSV keratitis [59]. IL-17-induced lymphangiogenesis has been reported in animal models of DED, particularly in chronic DED [55]. IL-17 secretion caused by desiccating stress induces corneal lymphangiogenesis through a VEGF-D/C-VEGFR3 signaling pathway, and IL-17 blockage suppresses lymphangiogenesis and the mRNA expression of VEGF-D/C in DED cornea [55]. Although the mechanism by which IL-17 induces VEGF-D/C remains unclear, pathways involving direct upregulation of VEGF-D by IL-17 receptor-expressing cells such as corneal epithelial and stromal cells and indirect upregulation of VEGF-C through IL-1β-mediated pathways are suspected [55,60]. However, IL-17 has recently been reported as a negative regulator of lymphangiogenesis during the resolution phase of Th17-mediated immune responses in a model of cholera toxin-mediated lung inflammation [61], suggesting that the effect of IL-17 on lymphangiogenesis may differ depending on the organ or situation.

## 2. Lymphatic Endothelial Cell (LEC) Markers

Known LEC markers PROX1, VEGFR-3, podoplanin, LYVE-1, and CD34 help to distinguish LV from the blood vascular endothelium. However, as there is no known definite marker(s) specifically expressed on LECs, it is not easy to differentiate LECs from other types of cells, including blood vascular endothelium, pericytes, and fibroblasts clearly [2]. Interestingly, LECs and VECs preferentially show homotypic interactions not just in vivo but also in vitro. Approximately 2% of transcribed genes are differentially expressed between LECs and VECs, and this difference may reflect their distinct in vivo functions [2,62,63,64]. Recent advances in next-generation sequencing (NGS) methods, as well as classical gene knockout or knockdown models, may reveal more specific conditional marker(s) for LEC and a comprehensive view of lymphatic biology. Here, we briefly review some key markers of LECs.

- PROX1
  In a knockout mouse model, *Prox1* deletion resulted in complete loss of LV. Although LEC buds from veins were found morphologically, they did not express LEC markers and failed to migrate further under *Prox1* knockdown conditions [65,66]. In accordance with the mouse model, *Prox1* overexpression in human VECs showed reduced expression of VEC-specific genes with the upregulation of LEC-specific genes [2,62].
- LYVE-1
  LYVE-1 may be the most widely used marker for LEC research. LYVE-1 is the first marker of LEC development, and interestingly, it is expressed in a polarized manner in the venous endothelium since early lymphatic development. In mammals, LYVE-1 is mainly expressed in lymphatic capillaries and is downregulated in large LVs [2,67]. The functional role of LYVE-1 in the regulation of lymphatic development during embryogenesis and lymphangiogenesis in adults remains unclear.
- Podoplanin
  Although podoplanin is widely used as an LEC marker, it is also highly expressed in various cells, including LECs, podocytes, keratinocytes, and alveolar cells in the lungs. Podoplanin knockout mice showed paw lymphedema and abnormal lymphatic function and pattern, which may reflect impaired LEC migration [68].
- VEGFR3 and VEGF-C/D: see Section 1.1
- Chemokine (C-C motif) ligand 21
  CCL21 is secreted by LECs but not by VECs [69] and interacts with the CC chemokine receptor 7 (CCR-7), which is expressed on the surface of immune cells. It works as a guide to the immune cells, mainly dendritic cells bearing antigens and homing from the tissues into the LVs and the secondary lymphatic organs; thus, it plays an important role in immunoregulatory and inflammatory processes. CCL21 has also been shown to enhance LN metastasis in CCR-7-expressing malignant melanoma cells [70].
- Desmoplakin
  Desmoplakin is a cytoplasmic anchor protein of LEC adherens junctions that connect intermediate filaments to the plasma membrane. Desmoplakin is not expressed in VECs [71].

## 3. Cytokines Promote or Inhibit Lymphangiogenesis in Various Disease Models

As described above, proinflammatory cytokines (e.g., IL-1β, IL-12, and IL-18) regulate mRNA transcription of *vegfc*, thereby indirectly affecting lymphatic vessel growth and function [47,72]. Chung and Dana [73] showed that IL-1β and TNF-α induce VEGF-C expression in the corneal micropellet model, promoting lymphangiogenesis [73]. Chronic ocular inflammation, DED, and type IV hypersensitivity induced severe ocular allergy are accompanied by increased lymphangiogenesis, correlating with increased expression of IL-1β and TNF-α acting via a dramatic increase in VEGF-C but not VEGF-D or FGF2 expression [74,75,76]. In diabetes, inflammation-induced TNF-α promotes lymphangiogenesis around islet cells, contributing to disease pathogenesis [1].

In contrast, anti-inflammatory cytokines negatively regulate lymphangiogenesis. IL-4 and -13 reportedly have anti-lymphangiogenic effects and impair LEC survival, proliferation, migration, and tube formation in vivo and in vitro [77,78]. However, specific cytokine-induced lymphangiogenesis is context-dependent. For example, TGF-β can induce or inhibit lymphangiogenesis [44,46].

## 4. Lymphangiogenesis in Ocular Surface Disease

### 4.1. Lymphangiogenesis in Allograft Rejection

**Figure 4:**
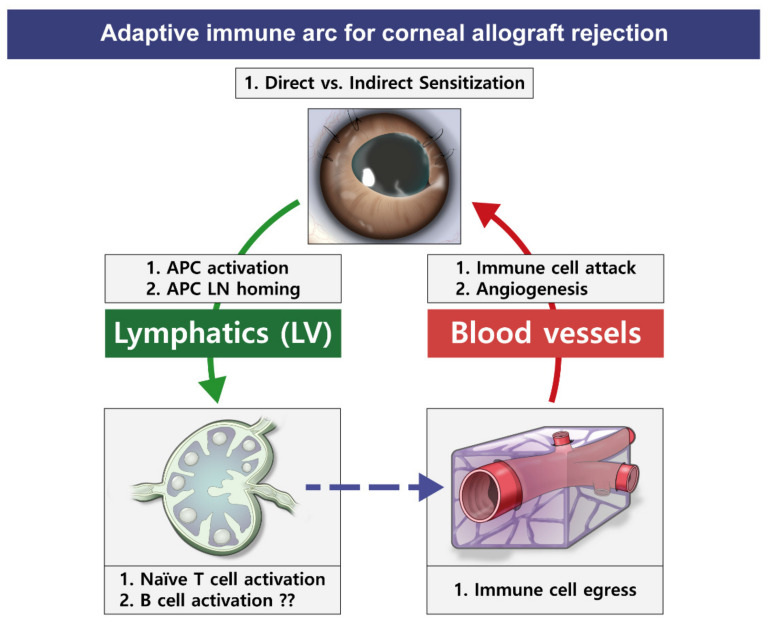
Adaptive immune arc for corneal allograft rejection. Antigen presenting cells (APCs) from donor corneal button (direct sensitization) or recipient bed (indirect sensitization) leave and migrate through lymphatic vessels into draining lymph nodes (LN). The T cells activated from the interaction between APCs and naive T cells then egress into peripheral blood and immune attack the donor cornea.

Ocular immune privilege is a phenomenon that is tightly bound to the avascularity of the cornea because the BVs and LVs constitute the afferent and efferent arms of an immune reflex arc, respectively (Figure 4) [79]. As an immune afferent arm of the corneal allograft, LV facilitates immune cell trafficking and transports antigen materials to regional LNs, accelerating the sensitization process in regional LNs. As an efferent arm, BVs facilitate the migration and infiltration of alloimmune cells into the donor cornea. Histologically, rejected corneal button and bed are characterized by a plethora of infiltrated immune cells, such as CD4+ or CD8+ T cells, macrophages, neutrophils, and natural killer cells. An increasing number of antigen-specific dendritic cells (DCs) in draining LNs arise only a few hours after transplantation [80]. These antigen-presenting cells in regional LNs can originate from the donor or host tissue. In this context, LVs rather than BVs toward the draining LNs determine the high-risk status of a corneal graft recipient. Therefore, the determination of LV development status in the cornea after transplantation may be a biomarker for allograft survival or rejection determination. However, unlike visible BVs, LVs are clinically invisible. Therefore, this invisibility has hampered their detection and the importance and functional role of LVs in allograft rejection and acceptance have been clinically disregarded. Through studies using recently discovered LEC markers, it is acknowledged both BVs and LVs play a role in allograft rejection and maintenance as the efferent and afferent arm of corneal alloimmunity, respectively. Table 1 summarizes the literature on corneal allografts published in the last decade.

Recently, microscopic optical coherence tomography (mOCT) [81] and intrastromal fluorescein dye injection techniques [82] have shown that LVs are visible in slit-lamp-based systems. Although both systems may be practically applicable for allograft research, determination of LV development in the clinical setting is not currently possible.

To determine the functional role of LV in allograft rejection, using alymphatic or prehem- and prelymph-angiogenic in vivo models, Dietrich et al. [83] transplanted cornea and found better graft survival in the alymphatic model than prelymphvascularized bed. Regenfuss and Cursiefen (same group of Reference [83]) [84] also reported that the lymphatic vasculature suggests a high-risk status of the recipient bed. In humans, LVs have also been found in rejected keratoplasty buttons. Diamond et al. found that podoplanin^hi^ LVs were distinct from BVs in failed human corneal grafts. Moreover, VEGFR-3 and LYVE-1 mRNA levels were found to be elevated [85]. Previously, our group also found LVs in the corneal button from pseudophakic bullous keratopathy and HSV-1 keratitis [86].

Although transplantation is the method of choice for completely reviving tissue function, graft rejection by alloimmunity may be the greatest barrier to transplantation success. As antigen presentation by professional dendritic cells (APCs) is the first step of alloimmune activation, many previous investigations have focused on preventing LVs from improving corneal allograft survival, using both medical and surgical interventions. Both angiogenesis and lymphangiogenesis are mainly driven by the VEGF system. Therefore, it is not unusual that there have been numerous studies on modulating VEGFs and their receptors to reduce angiogenesis and lymphangiogenesis to improve corneal allograft survival.

### 4.2. Lymphangiogenesis in Dry Eye Disease

**Figure 5:**
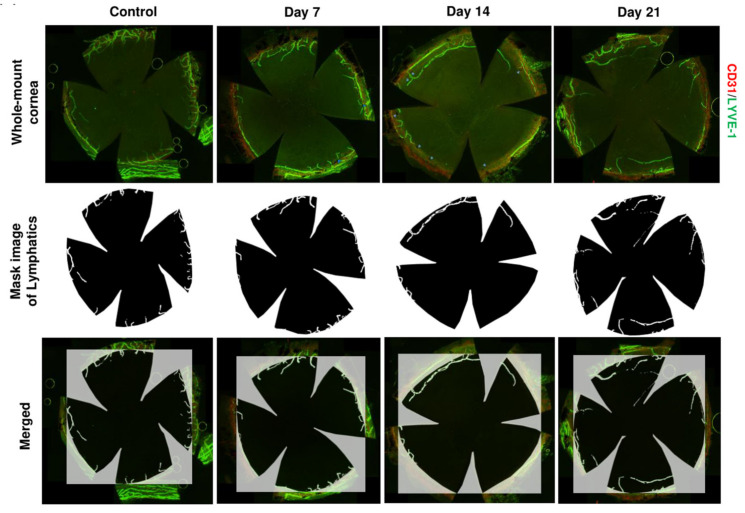
Lymphangiogenesis induced in the originally avascular cornea by housing mice under desiccating conditions. Representative immunofluorescence images of whole-mount cornea of the control group and dry eye group on days 7, 14, and 21 of dry eye induction in a controlled environmental chamber (upper set), the mask image of lymphatics automatically generated by the custom-designed macro of ImageJ (middle set), and merged whole-mount and mask images (lower set). The boundary of the cornea was decided considering the pigments (blue arrowheads), which were left at the place where the iris and ciliary body were attached. Corneas were immunostained with CD31 (red) and LYVE-1 (green) antibodies. (Courtesy of Lee et al., Ocul Surf. 2021 Jul 24;22:72–79. under the terms of the Creative Commons CC-BY license, Ref. [50]).

Traditionally, LVs and BVs have been thought to always co-exist and generate simultaneously, function as the afferent and efferent arms, respectively, of immune cell trafficking in most pathological conditions. Indeed, research on certain ocular inflammatory conditions, including infectious keratitis, chemical burns, and allograft rejections, has demonstrated that lymphangiogenesis and angiogenesis (new blood vessel generation from existing vessels) always occur together and cannot be separated. However, to investigate lymphangiogenesis more precisely, it is essential to develop lymphangiogenesis-only conditions. However, despite numerous trials, animal models or conditions that are widely accepted for “selective lymphangiogenesis” without angiogenesis have not yet been established. Chung and Dana [73] initially found lymphangiogenesis-dominant corneal micropocket assay conditions using 80 ng bFGF micropellet. However, because of the complicated method of micropellet preparation and loss of cytokine pellets during delicate micropellet implantation, the results were not always consistent. Furthermore, as some BVs still exist and sometimes grow enormously, the method could not distinguish LV from BVs. Interestingly, the cornea was found to generate LVs without BVs under desiccating stress [50,55,87,88,89] (Figure 5). Goyal and Dana [90] first found corneal lymphangiogenesis without angiogenesis in a mouse model of desiccated stress and the model was replicated in several subsequent studies [91]. Importantly, they found that corneal lymphangiogenesis was associated with increased activation of CD11b+ DCs (MHC-II+) in draining LNs, providing potential evidence that lymphangiogenesis in DED facilitates adaptive immune responses. In a subsequent study, anti-VEGF-C antibody reduced LVs and their caliber with CD11b+ cell recruitment in the cornea [87].

**Figure 6:**
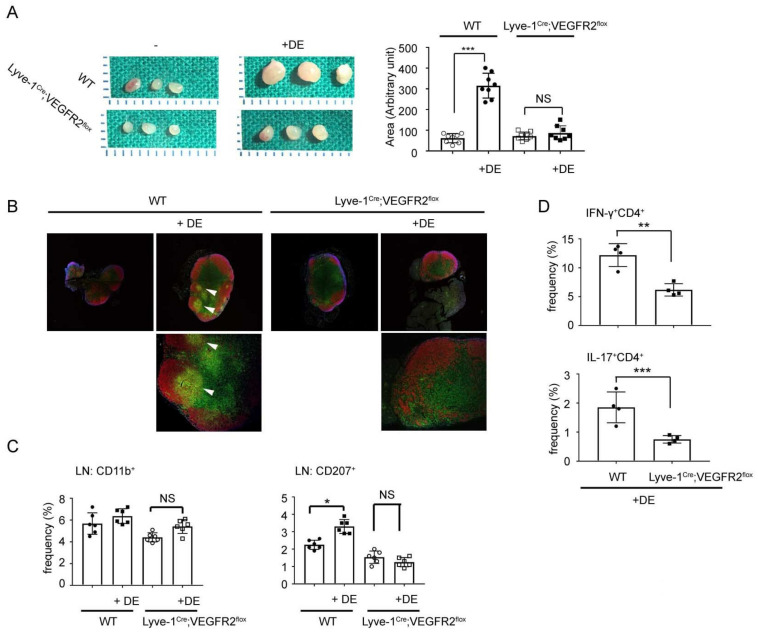
Morphohistological and cellular compositional changes of draining lymph nodes by dry eye induction in Lyve-1^Cre^;VEGFR2^flox^ mice. (**A**) Representative images of draining lymph nodes (LNs) from naïve and dry eye (DE)-induced mice. To determine LN size, at least three draining LNs from naïve and DE-induced female mice were measured using ImageJ software. Five mice, for a total of 15 LNs per group, were included in the calculation. (**B**) Confocal microscopy image of a draining LN section stained with CD3 (yellow) and B220 (red). White arrowheads indicate T and B cell mixed zone between corticomedullary junctions (magnification: 50× in upper row; 100× in lower row). (**C**,**D**) Flow cytometric analysis of CD11b^+^, CD207^+^, IFN-γ^+^CD4^+^, or IL-17^+^CD4^+^ cells obtained from draining LNs. Cell frequencies were measured with three independent experiments at 0 and 7 days after DE induction. Data are represented as means (bars) ± standard deviations (error bars) (***, *p* < 0.001; **, *p* < 0.01; *, *p* < 0.05, by one-way ANOVA with Dunnett’s post *hoc* test or independent *t*-test; NS, no statistical significance) (Ref. [92]).

Although the aforementioned study showed a glimpse of the functional role of LVs in DED, the mechanism and functional role of “selective lymphangiogenesis” without angiogenesis has not been established. Therefore, we designed a conditional knockout murine model, which reduced de novo synthesis of LVs using the LYVE-1^Cre^:VEGFR-2^flox^ system. In this model, we found that corneal erosion score, inflammatory cell infiltration, and pro-inflammatory cytokines were significantly reduced in the ocular surface, and the preservation of corneal nerves and reduced LN sizes was possible by inhibition of APC and T cell activation by prevention of corneal lymphangiogenesis [92] (Figure 6). We believe that our study and that by Dana and Goyal complements one another, and together, they provide strong evidence that corneal lymphangiogenesis facilitates immune cell activation, enhances pro-inflammatory cytokines, damages corneal nerves, and aggravates clinical scores [91]. Although lymphangiogenesis in the ocular surface is critical under DED conditions, LV growth proved more prominent in the lacrimal gland in the desiccated murine model from our previous studies [74,93] and communications with others (Chauhan, Sunil). Moreover, comparing ocular surfaces, protein biomarkers, and immune cell infiltrations were more abundant in the lacrimal gland than in the ocular surface and tears [94]. Therefore, further studies are necessary to fully elucidate DED-induced lymphangiogenesis in lacrimal glands.

The mechanism for selective lymphangiogenesis in DED-induced cornea is still largely unknown. Previously, thrombospondin-1, a well-known anti-angiogenic surface molecule in the corneal epithelium, was found to be an important antilymphangiogenic molecule [95]. In contrast, IL-1Ra and Th17/IL-17 were found to generate LVs in DE-induced mouse cornea. Recently, in the Hippo pathway, YAP/TAZ was found to be a negative regulator of corneal lymphangiogenesis. Deletion or hyperactivation of YAP/TAZ aggravated or attenuated pathological lymphangiogenesis in the mouse cornea by regulating PROX1 expression in LECs [96]. Min et al. found that the Dll4/NOTCH/HIF-1a axis plays an essential role in DED-induced lymphangiogenesis in the lacrimal gland. To understand DED-induced selective corneal and lacrimal gland lymphangiogenesis clearly, more sophisticated in vivo studies using conditional knock-out systems and human studies may be essential. Some essential studies on DE-induced lymphangiogenesis are summarized in Table 2.

### 4.3. Lymphangiogenesis in Other Ocular Diseases

In addition to keratoplasty and DED, lymphangiogenesis has been found in HSV-1 keratitis [97,98,99], allergic disease [75], bacterial keratitis [89], and corneal edema [100]. HSV-1 induced corneal lymphangiogenesis has been investigated for decades and is relatively well documented. HSV-infected corneal epithelium expressed VEGF-A with soluble VEGFR-1 reduction, which eventually induced lymphangiogenesis as well as angiogenesis [98]. Further, closer inspection of HSV-1 infected cornea reveals type I interferon and intact receptor system helps stop viral replication and keeps LV intact [101]. In addition, CD4+, CD8+ T cells, neutrophils, and natural killer cells have been found in HSV-1 infected cornea and their roles in HSV-1 keratopathy have been documented. However, the exact functional roles of these cells, as well as secretory factors induced by these cells (e.g., CXCL1, CXCL10) are still controversial [102]. Table 3 summarizes recent studies included in our review.

## 5. Inhibition of Lymphangiogenesis

### 5.1. Inhibition of VEGFs

VEGF-A has emerged as a principal cytokine responsible for both hemangiogenesis and lymphangiogenesis. Therefore, VEGF-A neutralization may directly or indirectly exert anti-angiogenic effects, inhibiting immune cell chemotaxis, especially mononuclear cells [103,104] and decreasing lymphangiogenesis [98,103]. However, as discussed above, VEGF-C/D are the primary cytokines for inhibiting lymphangiogenesis and bind with the high-affinity receptor VEGFR-3. Although many VEGF-A inhibitors have been developed and used clinically, there are currently no FDA-approved VEGF-C/D inhibitors.

Bevacizumab, which binds and neutralizes all VEGF-A isoforms, is widely used as an anti-angiogenic agent for cancer treatment and has also been reported to be successful in treating ocular neovascularization as off-label use. Dastjerdi et al. reported reduced corneal allograft rejection after topical or subconjunctival bevacizumab use in a murine allograft model [105]. In humans, it has been reported that subconjunctival/intrastromal injection or topical treatment leads to reduced new BVs in allograft rejection; however, all the studies were performed with a small case series [106,107,108]. Moreover, no human data exist to explain reduced lymphangiogenesis with VEGF-A neutralization in corneal allografts.

### 5.2. VEGFR-1/2 Inhibition

It is well known that VEGFR-1, which has the highest binding affinity for VEGF-A, showed strong chemotaxis for mono/macrophages under inflammatory conditions [104,109]. Chemotaxis for mononuclear cells under inflammatory conditions consequently results in lymphangiogenesis by releasing lymphangiogenic growth factors. Through soluble VEGFR-1 (sVEGFR-1)/Fc and sVEGFR-2/Fc chimera treatment, Hayashi et al. found significantly reduced new vessels in both treatment models. sVEGFR-1/Fc was found to impede wound healing and result in graft failure but not sVEGFR-2/Fc treatment [110]. They also found reduced donor-derived CD11c+ DCs, CD11b+ cell infiltration, and lymphangiogenesis. In addition, VEGFR-1 morpholinos, synthetic DNA oligonucleotides that can bind with RNA to block translation or alternative splicing, significantly improved graft survival by reducing lymphangiogenesis [111].

### 5.3. VEGFR-3 Inhibition

sVEGFR3 exists in the corneal epithelium and is known to be an important factor for inhibiting corneal vascularity [112]. Emani-Naeini et al. reported that sVEGFR-3 inhibited lymphangiogenesis, reduced IFN-γ+CD4+ cell infiltration in the corneal allograft, and improved survival [113]. Additionally, using both VEGFR-3 neutralizing antibody and integrin α5β1 inhibition, corneal allograft survival improved significantly and reduced lymphangiogenesis [83]. This study implicated that pre-existing LVs were important for graft survival because lymphangiogenesis inhibition severed the afferent arm of the immune response arc.

### 5.4. Non-VEGFs-Based Lymphangiogenesis Inhibition

In addition to VEGF-related studies, some other studies have shown improved corneal allograft survival by inhibiting lymphangiogenesis. Corneal collagen crosslinking (CXL) is usually used in ectatic corneal diseases to stabilize stromal tissue via the bonding of collagen fibers. Hou et al. were the first to report that CXL regressed preexisting BVs and LVs in a murine corneal allograft model. They found reduced inflammation, BVs, and LVs in TUNEL+ apoptotic cells [114]. As previously mentioned, the pre-existing vascular cornea is at high risk for allograft rejection. Therefore, if clinical trials confirm that CXL using riboflavin is an effective option for reducing LVs, the treatment before corneal allograft surgery will be a promising treatment option in the future. Photodynamic therapy (PDT) is designed as an anti-angiogenic strategy that utilizes a photosensitizer and light source for the inhibition of retinal blood vessels. In the cornea, Bucher et al. reported that PDT with verteporfin effectively and selectively regressed LVs without affecting mature BVs [115]. Interestingly, the same group showed that PDT using intravenous verteporfin lead to regression of both mature corneal BV and LVs in a time-dependent manner [116]. Lastly, fine needle diathermy (FND), which has been used to treat corneal angiogenesis, could reduce LV in mice. Interestingly, in a mouse model, pretreatment of the prevascularized high-risk eye with FND significantly improved allograft survival [117]. As FND has already been used clinically for corneal angiogenesis, previous studies that showed improved clinical results may also be relevant to reduce lymphangiogenesis. Recently, Hos and Cursiefen showed promising results in a clinical pilot study [118].

Blockade of the SP/NK1R system has also been reported as a possible target for suppressing corneal lymphangiogenesis. The anti-lymphangiogenic effects of anti-NK1Rs (lanepitant, befetupitant, fosaprepitant, and L733,060) and knockout of the pre-protachykinin A (*Tac1*) gene have been confirmed in animal models of alkali burn, suture-induced angiogenesis, and DED using whole-mount corneal staining for LEC markers [50,53,54,119]. However, although the beneficial effect of SP on corneal wound healing and its role in innate immunity against infection, there are several hurdles to be overcome for clinical application.

## 6. Conclusions

Although there has been a remarkable improvement in our understanding of lymphangiogenesis and advances in the regulation of corneal lymphatics, complete regulation of corneal lymphangiogenesis is still not possible, even in an in vivo model. LVs are distributed throughout the body, but the molecular mechanism of lymphangiogenesis is different in each tissue and organ and varies under different pathological conditions. In addition, corneal lymphangiogenesis is closely linked to hemangiogenesis and shares partially distinct and partially identical molecular mechanisms.

In terms of lymphangiogenesis inhibition, as corneal LVs are not found under normal conditions, inhibition of corneal lymphangiogenesis is one approach to restore a “pathological” condition to a “normal physiological” cornea. Although research has been conducted for several decades, it is still not possible to effectively remove LVs or reduce lymphangiogenesis in the cornea. Topical application of anti-VEGF-A, VEGF-C, or VEGFR-3 has been reported to promote corneal allograft survival by reducing lymphangiogenesis in a murine model. Based on the success of in vivo research, human trials for reducing corneal lymphangiogenesis have started and show promising results despite the small number of pilot studies [116,118].

We also suggest future studies that are essential to understand ocular lymphangiogenesis more clearly, as well as to make clinical improvements in vasculogenic corneal disease, including allograft rejection and DED (Table 4). When these research and clinical needs are met, we may significantly improve the clinical outcome of high-risk corneal allograft, regulate DED more easily, and improve the morbidity of incurable ocular surface disease.

## Figures and Tables

**Table 1:** Recent literature on corneal allograft rejection and lymphangiogenesis.

Authors	Title	Journal	PMID
Schönberg A	Immunomodulatory Strategies Targeting Dendritic Cells to Improve Corneal Graft Survival	J Clin Med. 2020 Apr 28;9(5):1280. doi:10.3390/jcm9051280.	32354200
Hos D	Immune reactions after modern lamellar (DALK, DSAEK, DMEK) versus conventional penetrating corneal transplantation	Prog Retin Eye Res. 2019 Nov;73:100768. doi:10.1016/j.preteyeres.2019.07.001. Epub 2019 Jul 3.	31279005
Hori J	Immune privilege in corneal transplantation	Prog Retin Eye Res. 2019 Sep;72:100758. doi:10.1016/j.preteyeres.2019.04.002. Epub 2019 Apr 20.	31014973
Yu T	The atypical chemokine receptor-2 does not alter corneal graft survival but regulates early stage of corneal graft-induced lymphangiogenesis	Graefes Arch Clin Exp Ophthalmol. 2018 Oct;256(10):1875–1882. doi:10.1007/s00417-018-4070-1. Epub 2018 Jul 27.	30054731
Le VNH	Fine Needle-Diathermy Regresses Pathological Corneal (Lymph)Angiogenesis and Promotes High-Risk Corneal Transplant Survival	Sci Rep. 2018 Apr 9;8(1):5707. doi:10.1038/s41598-018-24037-3.	29632336
Su W	Pharmacological inhibition of caspase-8 suppresses inflammation-induced lymphangiogenesis and allograft rejection in the cornea	J Allergy Clin Immunol. 2018 Jul;142(1):290–294.e9. doi:10.1016/j.jaci.2018.02.005. Epub 2018 Mar 2.	29477723
Zhong W	Angiogenesis and lymphangiogenesis in corneal transplantation-A review	Surv Ophthalmol. 2018 Jul-Aug;63(4):453–479. doi:10.1016/j.survophthal.2017.12.008. Epub 2017 Dec 27.	29287709
Hou Y	Photodynamic Therapy Leads to Time-Dependent Regression of Pathologic Corneal (Lymph) Angiogenesis and Promotes High-Risk Corneal Allograft Survival	Invest Ophthalmol Vis Sci. 2017 Nov 1;58(13):5862–5869. doi:10.1167/iovs.17-22904.	29145577
Zhang L	Angiopoietin-2 Blockade Promotes Survival of Corneal Transplants	Invest Ophthalmol Vis Sci. 2017 Jan 1;58(1):79–86. doi:10.1167/iovs.16-20485.	28061513
Chen WS	Pathological lymphangiogenesis is modulated by galectin-8-dependent crosstalk between podoplanin and integrin-associated VEGFR-3	Nat Commun. 2016 Apr 12;7:11302. doi:10.1038/ncomms11302.	27066737
Schöllhorn L	Thrombospondin-1 as a Regulator of Corneal Inflammation and Lymphangiogenesis: Effects on Dry Eye Disease and Corneal Graft Immunology	J Ocul Pharmacol Ther. 2015 Sep;31(7):376–85. doi:10.1089/jop.2015.0020. Epub 2015 Jul 8.	26154823
Seo Y	Expression of Lymphangiogenic Markers in Rejected Human Corneal Buttons after Penetrating Keratoplasty	Curr Eye Res. 2015 Sep;40(9):902–12. doi:10.3109/02713683.2014.969809. Epub 2014 Oct 20.	25330436
Emami-Naeini P	Soluble vascular endothelial growth factor receptor-3 suppresses allosensitization and promotes corneal allograft survival	Graefes Arch Clin Exp Ophthalmol. 2014 Nov;252(11):1755–62. doi:10.1007/s00417-014-2749-5. Epub 2014 Aug 5.	25091513
Hos D	Lymphatic vessels in the development of tissue and organ rejection	Adv Anat Embryol Cell Biol. 2014;214:119–41. doi:10.1007/978-3-7091-1646-3_10.	24276891
Flynn TH	The effect of perioperative allergic conjunctivitis on corneal lymphangiogenesis after corneal transplantation	BrJ Ophthalmol. 2011 Oct;95(10):1451–6. doi:10.1136/bjo.2010.201939.Epub 2011 Jun 7.	21653212
Dietrich T	Cutting edge: lymphatic vessels, not blood vessels, primarily mediate immune rejections after transplantation	J Immunol. 2010 Jan 15;184(2):535–9. doi:10.4049/jimmunol.0903180. Epub 2009 Dec 16.	20018627
Maruyama K	The maintenance of lymphatic vessels in the cornea is dependent on the presence of macrophages	Invest Ophthalmol Vis Sci. 2012 May 31;53(6):3145–53. doi:10.1167/iovs.11-8010.	22511631

**Table 2:** Recent literature on dry eye disease and lymphangiogenesis.

Author	Title	Journal	PMID
Seo, Y	Activation of HIF-1α (hypoxia inducible factor-1α) prevents dry eye-induced acinar cell death in the lacrimal gland	Cell death & disease 2014, 5, e1309. doi:10.1038/cddis.2014.260.	24967971
Goyal, S	Blockade of prolymphangiogenic vascular endothelial growth factor C in dry eye disease	Arch ophthalmol 2012, 130, 84–89. doi:10.1001/archophthalmol.2011.266.	21911653
Chennakesavalu, M	Corneal lymphangiogenesis as a potential target in dry eye disease–a systematic review	Sur ophthalmol 20212021 Mar 31;S0039-6257(21)00080-1. doi:10.1016 /j.survophthal.2021.03.007	33811911
Goyal, S	Evidence of corneal lymphangiogenesis in dry eye disease: a potential link to adaptive immunity?	Arch ophthalmol 2010, 128, 819–824, doi:10.1001/archophthalmol.2010.124.	20625040
Lee, SJ	Corneal lymphangiogenesis in dry eye disease is regulated by substance P/neurokinin-1 receptor system through controlling expression of vascular endothelial growth factor receptor 3	Ocul surf 2021, 22, 72–79. doi:10.1016/j.jtos.2021.07.003.	34311077
Min JH	Activation of Dll4/Notch Signaling and Hypoxia-Inducible Factor-1 Alpha Facilitates Lymphangiogenesis in Lacrimal Glands in Dry Eye	PLoS One. 2016 Feb 1;11(2):e0147846. doi:10.1371/jounal.pone.0147846. eCollection 2016.	26828208
Ji YW	Corneal lymphangiogenesis facilitates ocular surface inflammation and cell trafficking in dry eye disease	Ocul Surf. 2018 Jul;16(3):306–313 doi:10.1016/j.jtos.2018.03.008. Epub 2018 Mar 27.	29601983
Okanobo A	Efficacy of topical blockade of interleukin-1 in experimental dry eye disease	Am J Ophthalmol 2012, 154, 63–71. doi:10.1016/j.ajo.2012.01.034.	22541929

**Table 3:** Recent literature on keratitis, ocular allergy, and lymphangiogenesis.

Authors	Title	Journal	PMID
Narimatsu A.	Corneal lymphangiogenesis ameliorates corneal inflammation and edema in late stage of bacterial keratitis	Sci Rep. 2019 Feb 27;9(1):2984. doi:10.1038/s41598-019-39876-x.	30814667
Gurung HR	Fibroblast growth factor-2 drives and maintains progressive corneal neovascularization following HSV-1 infection	Mucosal Immunol. 2018 Jan;11(1):172–185. doi:10.1038/mi.2017.26. Epub 2017 Apr 5.	28378806
Gurung HR	Cornea lymphatics drive the CD8+ T-cell response to herpes simplex virus-1	Immunol Cell Biol. 2017 Jan;95(1):87–98. doi:10.1038/icb.2016.80. Epub 2016 Aug 31.	27577867
Lee HS	Involvement of corneal lymphangiogenesis in a mouse model of allergic eye disease	Invest Ophthalmol Vis Sci. 2015 May;56(5):3140–8. doi:10.1167/iovs.14-16186.	26024097
Park PJ	Corneal lymphangiogenesis in herpetic stromal keratitis	Surv Ophthalmol. 2015 Jan–Feb; 60(1):60–71. doi:10.1016/j.survophthal.2014.06.001. Epub 2014 Jun 10.	25444520
Wuest TR	VEGF-A expression by HSV-1-infected cells drives corneal lymphangiogenesis	J Exp Med. 2010 Jan 18;207(1):101–15. doi:10.1084/jem.20091385. Epub 2009 Dec 21.	20026662
Suryawanshi A	IL-17A differentially regulates corneal vascular endothelial growth factor (VEGF)-A and soluble VEGF receptor 1 expression and promotes corneal angiogenesis after herpes simplex virus infection	J Immunol 2012, 188, 3434-3446. doi:10.4049/jimmunol.1102602.	22379030

**Table 4:** Unmet needs to be investigated for ocular surface lymphangiogenesis.

Title	Remarks
Cross-talk between infiltrating myeloid cells, T cells, and LECs	Determine the role of infiltrating myeloid cells at the early stage of lymphangiogenesis
Effectiveness of VEGF inhibitors in allograft survival and lymphangiogenesis	Large scale clinical research for VEGF inhibitors is needed
Transcriptome analysis of lymphangiogenesis at the single-cell level	Cell-cell interaction is more clearly defined with scRNA-seq at each step of lymphangiogenesis
Proteomic analysis of lymphangiogenesis in each pathologic condition	OMICs study is essential to clearly understand and define druggable targets for lymphangiogenesis
Role of lymphangiogenesis in incurable ocular surface disease (e.g., OCP, GVHD)	Studies of lymphangiogenesis on severe ocular vascular diseases are sparse
Role of cells residing on the ocular surface and immune cells	Determine the role of corneal epithelium, keratocyte, and endothelial cells in lymphangiogenesis
Effective molecular target for developing drugs	Besides VEGFs, more effective and durable targets for drug development should be investigated

LEC, lymphatic endothelial cell; VEGF, vascular endothelial growth factor; OCP, ocular cicatricial pemphigoid; GVHD, graft-versus-host disease.

## Data Availability

Not applicable.
